# Medicinal plants extracts affect virulence factors expression and biofilm formation by the uropathogenic *Escherichia coli*

**DOI:** 10.1007/s00240-012-0499-6

**Published:** 2012-08-23

**Authors:** Dorota Wojnicz, Alicja Z. Kucharska, Anna Sokół-Łętowska, Marta Kicia, Dorota Tichaczek-Goska

**Affiliations:** 1 Department of Biology and Medical Parasitology, Wrocław Medical University, Mikulicza-Radeckiego 9, 50-367 Wrocław, Poland; 2 Department of Fruit and Vegetables and Cereals Technology, Wrocław University of Environmental and Life Sciences, Norwida 25, 50-375 Wrocław, Poland

**Keywords:** UPEC, Plant extracts, Biofilm, Virulence factors

## Abstract

Medicinal plants are an important source for the therapeutic remedies of various diseases including urinary tract infections. This prompted us to perform research in this area. We decided to focus on medicinal plants species used in urinary tract infections prevention. The aim of our study was to determine the influence of *Betula* *pendula*, *Equisetum arvense*, *Herniaria glabra*, *Galium odoratum*, *Urtica dioica*, and *Vaccinium vitis*-*idaea* extracts on bacterial survival and virulence factors involved in tissue colonization and biofilm formation of the uropathogenic *Escherichia coli* rods. Qualitative and quantitative analysis of plant extracts were performed. Antimicrobial assay relied on the estimation of the colony forming unit number. Hydrophobicity of cells was established by salt aggregation test. Using motility agar, the ability of bacteria to move was examined. The erythrocyte hemagglutination test was used for fimbriae P screening. Curli expression was determined using YESCA agar supplemented with congo red. Quantification of biofilm formation was carried out using a microtiter plate assay and a spectrophotometric method. The results of the study indicate significant differences between investigated extracts in their antimicrobial activities. The extracts of *H. glabra* and *V. vitis*-*idaea* showed the highest growth-inhibitory effects (*p* < 0.05). Surface hydrophobicity of autoaggregating *E. coli* strain changed after exposure to all plant extracts, except *V. vitis*-*idaea* (*p* *>* 0.05). The *B. pendula* and *U. dioica* extracts significantly reduced the motility of the *E. coli* rods (*p* < 0.05). All the extracts exhibited the anti-biofilm activity.

## Introduction

Urinary tract infections (UTIs) are among the most common bacterial infectious diseases in human population. *E. coli* are the most predominant pathogens responsible for 80–90 % of community-acquired and 30–50 % of hospital-acquired UTIs [1]. Uropathogenic *E. coli* strains are equipped with a particular set of virulence determinants allowing them to colonize distinct sites in the urinary system. Hydrophobic cell surface, fimbriae P, curli fiber, ability to move allow them to successfully initiate infections. Bacterial cells after initial attachment to host tissues begin to grow and spread as a monolayer on the surface to form microcolonies that can disaggregate or create biofilm. *E. coli* biofilms are frequently described for catheter-associated, chronic and recurring UTIs. These structures protect the bacteria against the mechanical flow of urine, host and antibiotics [2, 3].

It is well known that herbal remedies are used by different human cultures since 1,000 of years. Some of those plant natural products are essential in prevention and treatment of UTIs. The most commonly known in this field are cranberry products; however, antibacterial properties of many other plants are also well known [4]. In the present study, we focused on leaf extracts of plants used traditionally in prevention of UTIs. Up to our knowledge, reports describing their antibacterial activities, especially against *E. coli* and urinary tract infections induced by these bacteria are highly limited and this fact prompted us to perform current study.

The purpose of this study was to evaluate the activity of *Betula* *pendula* (silver birch), *Equisetum arvense* (common horsetail), *Herniaria glabra* (smooth rupturewort), *Galium odoratum* (sweet woodruff), *Urtica dioica* (common nettle), and *Vaccinium vitis*-*idaea* (lingonberry) leaves extracts against uropathogenic *E. coli* rods as well as their impact exerted on virulence factors and biofilm formation.

## Materials and methods

### Plant materials

Six plant species commonly used in folk medicine in Poland were selected. Herbs were purchased from two herbs confectioning factories: “FLOS”, general partnership (Mokrsko, Poland) with marketing authorization numbers as follows: *H. glabra*—13736, *G. odoratum*—13561, and “KAWON-HURT”, general partnership (Gostyń, Poland) with marketing authorization numbers as follows: *B. pendula*—IL-3332/LN, *V. vitis*-*idaea*—IL-3333/LN, *E. arvense*—IL-3347/LN, *U. dioica*—R/2183.

### Preparation of extracts

Purchased dry herbs were ground into powder in an electric blender. 20 g of each leaf powder was dissolved in 180 mL of distilled water in a glass bottles, heated to 85 °C in a water bath and kept at this temperature with shaking for 8 h. After cooling, the liquid was filtered through the Whatman No. 1 filter paper. The filtrates were then condensed and dried in smaller glass bottles at 37 °C for 48 h. Then, the dried extracts were dissolved in distilled water to obtain concentrations ranging from 0.125 to 20.0 mg/mL.

### UPLC–Q-TOF–MS conditions

Compounds identification was performed on an Acquity ultra-performance liquid chromatography (UPLC) system coupled with a quadrupole-time of flight (Q-TOF) MS instrument (UPLC/Synapt Q-TOF MS, Waters Corp., Milford, MA, USA) with an electrospray ionization (ESI) source. Separation was achieved on a Acquity™ BEH C_18_ column (100 mm × 2.1 mm i.d., 1.7 μm; Waters). Detection wavelengths were set at 254, 280, 320, 380 and 520 nm. Mobile phase was a mixture of 4.5 % formic acid (A) and acetonitrile (B). The gradient program was as follows: initial conditions—99 % (A), 12 min—75 % (A), 12.5 min—100 % (B), 13.5 min—99 % (A). The flow rate was 0.45 mL/min and the injection volume was 5 μL. The column was operated at 30 °C. The major operating parameters for the Q-TOF MS were set as follows: capillary voltage 2.0 kV, cone voltage 45 V, cone gas flow of 11 L/h, collision energy 50 eV, source temperature 100 °C, desolvation temperature 250 °C, collision gas, argon; desolvation gas (nitrogen) flow rate, 600 L/h; data acquisition range, *m*/*z* 100–1,000 Da; ionization mode, negative. The data were collected by Mass-Lynx™ V 4.1 software.

### Bacterial strain

*Escherichia coli* clinical strain was isolated from the urine of patient with pyelonephritis, hospitalized in the Academic Hospital in Wrocław. The species affiliation of the examined strain was confirmed using the API-20E test kit (BioMérieux, Warsaw, Poland). The strain was maintained on Mueller–Hinton agar slopes (Oxoid) at 4 °C.

### Phylogenetic classification and virulence-associated genes carriage

The presence of selected nucleotide sequences was verified by PCR on total DNA isolated from bacterial overnight culture using GeneMATRIX Bacterial & Yeast Genomic DNA Purification Kit (EURx, Poland). All PCR analyses were performed using DreamTaq™ DNA polymerase (Fermentas, Germany). Phylogenetic group was determined using primers specific for two genes (*chuA* and *yjaA*) and an anonymous DNA fragment (*TspE4.C2*) according to the method of Clermont et al. [5]; however, instead multiplex PCR, *yjaA* sequence was amplified separately. Strain was screened for the presence of adhesins (*papC*, *sfa*, *afa*, *csgA*), siderophore (*aer*), toxins (*hlyA*, *cnf1*) and biofilm-related genes (*luxS*, *mcbA*, *mqsR*, *sdiA*, and *ant43*). Sequence coding for 16SrRNA was used as a positive control. The characteristics of all used primers, as well as amplicons length, are listed in Table 1.

**Table 1:** Primer sequences used in PCR

Gene	Primer name	Sequence (5′–3′)	Amplicon size (bp)	Reference or gene bank accession no (genome region)
*chuA*	ChuA.1	GACGAACCAACGGTCAGGAT	279	[5]
	ChuA.2	TGCCGCCAGTACCAAAGACA		
*yjaA*	YjaA.1	TGAAGTGTCAGGAGACGCTG	211	[5]
	YjaA.2	ATGGAGAATGCGTTCCTCAAC		
*TspE4.C2*	TspE4C2.1	GAGTAATGTCGGGGCATTCA	152	[5]
	TspE4C2.2	CGCGCCAACAAAGTATTACG		
*papC*	pap1	GACGGCTGTACTGCAGGGTGTGGCG	328	[6]
	pap2	ATATCCTTTCTGCAGGGATGCAATA	336	
	pap3	GCAACAGCAACGCTGGTTGCATCAT		
	pap4	AGAGAGAGCCACTCTTATACGGACA		
*sfa*	sfa1	CTCCGGAGAACTGGGTGCATCTTAC	410	[6]
	sfa2	CGGAGGAGTAATTACAAACCTGGCA		
*afa*	afa1	GCTGGGCAGCAAACTGATAACTCTC	750	[6]
	afa2	CATCAAGCTGTTTGTTCGTCCGCCG		
*csgA*	csgAF	GTAGCAGCAATTGCAGCAATCG	383	AE014075
	csgAR	TTAGATGCAGTCTGGTCAACAG		(1247944..1248402)
*aer*	aer1	TACCGGATTGTCATATGCAGACCG	602	[6]
	aer2	AATATCTTCCTCCAGTCCGGAGAAG		
*hlyA*	hlyA1.10f	GCTGCAAATAAATTGCACTCAG	665	[7]
	hlyA2.10r	CCCTGCACCGATATTATCAAG		
*cnf1*	cnf1	AAGATGGAGTTTCCTATGCAGGAG	498	[6]
	cnf2	CATTCAGAGTCCTGCCCTCATTATT		
*ant43*	ant43_F	TGGCACCATCAGCCTGCGTG	127	AE014075
	ant43_R	CGTACCACTGTTGCCGGCGT		(1225454..1228729)
*luxS*	luxS_F	CGGCAGCCCATTGGCGAGAT	178	AE014075
	luxS_R	TGAACACCCCGCATGGCGAC		(3096814..3097329)
*mcbA*	mcbA_F	CGCCTTGTTCGCGCGCTTTT	138	NC_000913
	mcbA_R	TCACGGCTTATGCCGCGCAA		(841019..841279)
*mqsR*	mqsR_F	GCCTGTAACAAGCCTGGGTCTGT	187	U00096
	mqsR_R	TGTCAATGCCGGGCAAGTTCGT		(3166270..3166566)
*sdiA*	sdiA_F	ATGGTACCGGGTGGCGGACA	130	AE014075
	sdiA_R	TGGCGTCGCACGATGCTGTT		(2144786..2145520)
*16srRNA*	rRNA16SF	AGAGTTTGATCATGGCTCAG	919	[8]
	rRNA16SR	CCGTCAATTCATTTGAGTTT		

### PCR products visualization and analysis

PCR amplification of the DNA was confirmed by running 20 μL of the PCR products on a 2 % agarose gel. Gel images were visualized and analyzed using the Quantity One system (Bio-Rad).

### Antibacterial activity of plant extracts

The antimicrobial activity of plant extracts was determined as described below. Briefly, the strain was grown overnight, and then bacterial cells were transferred to fresh Mueller–Hinton broth (MHB, BIOCORP, Warsaw, Poland) and incubated at 37 °C for 30 min. Following incubation, the bacterial cells were centrifuged (4,000 rpm for 20 min) and suspended in phosphate-buffered saline (PBS) to reach the final density 0.5 in McFarland scale. Bacterial suspension and plant extracts were mixed together to obtain following concentrations: 0.125, 0.25, 0.5, 1.0, 5.0, 10.0, 15.0, and 20.0 mg/mL of extract in sample. All samples were incubated at 37 °C for 24 h, then diluted and cultured on nutrient agar plates (BIOMED, Warsaw, Poland). After 24-h incubation at 37 °C, the number of colony forming units (c.f.u.) was counted. Control sample contained no plant extracts was taken as 100 % survival. The experiment was repeated three times. In each experiment, six repeats for control as well as examined, samples were taken.

### Effect of plant extracts on hydrophobicity of bacterial cells

Bacterial cells were incubated with plant extracts for 24 h at 37 °C. After incubation, they were washed three times in PBS. After last centrifugation, samples were diluted to obtain final optical density (measured at 470 nm) of 1.0. Untreated bacterial strain was assessed as a control. The salt aggregation test (SAT) of ammonium sulfate was used [9]. The control and treated suspensions (20 μL) were mixed with a series of dilutions of ammonium sulfate (20 μL) ranging from 0 to 3.2 M. The lowest concentration of ammonium sulfate at which bacterial aggregation was visible was determined. Each test was repeated three times. Based on the SAT values, the bacterial cell surface was classified as: <0.2 M—very strong hydrophobic, 0.4–1.0 M—strong hydrophobic, 1.2–1.6 M—hydrophobic, >1.8 M—hydrophilic.

### Effect of plant extracts on swimming motility

Bacterial cells were incubated with plant extracts and washed as described in previously. The final density of bacterial suspension was adjusted to 0.5 in McFarland scale. 10 μL of suspension was inoculated onto motility plates (1 % tryptone, 0.25 % NaCl, and 0.3 % agar). The plates were incubated at 37 °C for 24 h and the diameters of the swimming zone were measured [10]. Presented results are the mean of three experiments. In each experiment, four repeats for control as well as examined samples were taken.

### Effect of plant extracts on hemagglutination and expression of P fimbriae

Assays were performed on each strain grown overnight with plant extracts. After washing thrice in PBS, the final density of bacterial suspension was adjusted to 0.5 in McFarland scale. P fimbriae expression was confirmed by the hemagglutination of 3 % erythrocytes from human with blood group 0 in the presence or absence d-mannose [11]. The experiment was repeated three times.

### Effect of plant extracts on curli expression

Effect of plant extracts on curli expression was assessed according to Hammar et al. [12]. Bacterial suspension was prepared as described in paragraph devoted to hydrophobicity determination. 10 μL of suspension was inoculated onto plate containing YESCA agar supplemented with congo red (CRI) and the same subMICs. Curli-producing *E. coli* bound congo red dye and formed red colonies, whereas curli-negative bacteria formed white colonies. Control culture contained no plant extracts. The experiment was repeated three times.

### Biofilm formation assay and quantification

The capacity to form biofilms was assayed in microtiter plates essentially as described by O’Toole and Kolter [13] with slight modification. Briefly, cells were initially grown for 24 h in MHB at 37 °C. Subsequently, 150 μL overnight culture was added to 96-well polystyrene microtiter plates and incubated for 24 h at 37 °C. Unattached bacterial cells were then removed from the culture medium, and the biofilm was stained with 0.1 % (w/v) crystal violet for 15 min (this dye stains the cells but not the polystyrene). The excess crystal violet dye was washed out, and this was followed by washing the samples three times with distilled water. To release the dye, 200 μL 96 % ethanol was added to the wells. Subsequently, 125 μL sample was transferred to another well, and the optical density (OD) was measured at 495 nm using a plate reader (ANALCO-GBG STAT-FAX 2100). In each plate, four wells were used as blanks containing MHB medium. On the basis of ODs of bacterial biofilms, *E. coli* strains were classified into four categories [14]. The cut-off OD (ODc) was defined as three standard deviations (SD) above the mean OD of the negative control. Strains were classified as follows: OD ≤ ODc no biofilm producer, ODc < OD ≤ 2 × ODc weak biofilm producer, 2 × ODc < OD ≤ 4 × ODc moderate biofilm producer; 4 × ODc < OD strong biofilm producer. In our study the ODc value was 0.003.

### Effect of plant extracts on biofilm formation

Effect of plant extracts on biofilm formation was performed according to Di Bonaventura et al. [15]. Due to the smallest differences in survival of treated bacteria, the extracts concentrations 0.125 mg/mL were used in this experiment. Samples were prepared in microtiter plate wells by adding the appropriate volume of plant extract to 200 μL of MHB containing 20 μL of culture of bacteria (0.5 in McFarland scale). After 1–10 days of incubation, biofilm formation was measured as described in the previous section. Plant extract-free medium was used as a control. The results are given as mean values from three separate experiments. In each experiment, seven repeats for control as well as examined samples were taken.

### Statistical analysis

The differences in growth, motility and biofilm formation between rods exposed to plant extracts and unexposed were analyzed by a parametric *t* test for independent samples. Non-parametric Chi square test was used to correlate the concentrations of tested plant extracts and cell surface hydrophobicity. All tests were analyzed at the significance level *p* < 0.05 using Statistica 7.1.

## Results

### Qualitative and quantitative analysis of plant extracts

Identification of compounds was performed on the basis accurate mass searching, fragmentation analysis (MS/MS), comparison of accurate mass and matching of MS/MS pattern with standards and with data published in literature. The identified compounds and their LC/MS data are shown in Table 2. In the extract of *B. pendula*, 3,4′-dihydroxypropiophenone-3-β-d-glucoside (DHPPG), belonging to the group of propiophenone derivatives, was the main constituent. The other components present in significant amounts in this extract were quercetin derivatives belonging to flavonols (quercetin-3-galactoside, quercetin-3-glucuronide). Caffeic acid derivatives and *p*-coumaric acid derivatives were also identified. In the extract of *E. arvense*, three flavonols (quercetin dihexoside, kaempherol dihexoside, kaempherol-dirhamnosyl-hexoside) and four phenolic acids (protocatechuic, caftaric, ferulic, caffeic acids) were detected. The predominant compounds are caftaric acid and its derivatives (dimer and hexoside). In the extract of *H. glabra*, we found caffeoylquinic and feruloylquinic isomers, flavonols (quercetin, kaempherol and isorhamnetin derivatives) and iridoids. Phenolic acids (protocatechuic acid, caffeoylquinic isomers), flavonols (quercetin and kaempherol derivatives) and iridoids were the main compounds of *G. odoratum* extract. The dominant components of the *U. dioica* extract were phenolic acids (protocatechuic, ferulic, *p*-coumaric, and dicaffeoylquinic acids). Flavonols (quercetin derivatives), phenolic acids (derivatives of caffeoylquinic, caffeoyl-hexose-hydroxyphenol and coumaroyl-hexose-hydroxyphenol acids), procyanidins (A and B dimmers) and iridoids were the three most dominant compounds extracted from *V. vitis*-*idaea*.

**Table 2:** Compounds identified in plant extracts by using negative ions in LC–MS and MS/MS

Parent ion [M−H]^−^ (*m*/*z*)	Daughter ion MS/MS (*m*/*z*)	Compound	*B. pendula*	*E. arvense*	*H. glabra*	*G. odoratum*	*U. dioica*	*V. vitis*-*idaea*
Flavonols and derivatives								
269.1342		Apigenin derivative				x		
433.065	300.0171/271.0211/255.0303/179.0020	Quercetin-3-arabinopyranoside	x					x
433.1033	300.049	Quercetin-xyloside	x					x
447.0743	301.0319/300.0207	Quercetino-3-rhamnoside	x					x
463.0887	30.0319/300.0313	Quercetin-glucoside	x	x				
463.0931	300.0242/301.0354	Quercetin-galactoside						x
477.1022	301.0354	Quercetin-glucuronide	x					
477.1263	175.0372/301.0273/300.0348	Quercetin derivative						x
591.1436	529.1354/489.1132/447.1003/301.0461/300.0242	Quercetin-3-*O*-(4 bis-3-hydroxy-3-methylglutaryl)-α-rhamnoside						x
593.1606	285.0429	Kaempherol ramnohexoside				x		
609.1331	447.0916/285.0083	Kaempherol diglycoside		x				
609.1533	463.0931/301.0319	Rutin			x	x		x
623.1342	497.1168/315.0440/107.4882	Isorhamnetin rhamnose-hexose			x			
625.1591	463.0623/301.0176	Quercetin dihexoside		x				
737.1927	596.1407/284.0348	Hexoside-rhamnoside kaempferol and hydroxyferulic acid derivative			x			
755.3019	593.2354/447.0786/285.0498	Kaempherol-di-rhamnosyl-hexoside		x		x		
771.2246	609.1735/285.0049	Kaempherol-trihexoside		x				
771.3156	609.2391/463.1460/301.0603	Quercetin-hexoside-rhamnoside-hexoside				x		
Flavan-3-ols and procyanidins								
289.0688	245.0659/165.0118/137.0206/125.0218	Epicatechin						
289.0827	245.0787/203.0635/165.0065	Catechin						
575.1207	539.0969/449.0889/407.0727/289.0688/285.0325	Dimer procyanidin A						x
577.1201	407.0645/289.0758	Dimer procyanidin B						x
577.1594	289.0584/245.0370/125.0241	Dimer procyanidin B						x
577.1933	289.0897	Dimer procyanidin B						x
863.163	711.1367/693.1317/573.0953/451.1297/411.0632/289.0723	Trimer procyanidin A/B2						x
Iridoids								
389.0818	271.0616/124.9967/107.9938	Iridoide						x
389.0858	217.0116/198.9950/155.0041	Iridoide				x		
389.0939	191.0100/147.0193	Aucubioside				x		
389.0939	209.0213/183.0417/165.0302	Iridoide				x		
499.1633	337.1049/235.0395	Iridoide			x			
535.1362	371.1109/329.1297/311.0642/191.0326/163.0406	Coumaroyl iridoide						x
553.1731	389.11	Iridoide						x
Phenolic acids								
153.0027	109.0022/116.8969	Protocatechuic acid		x		x		
163.038	119.0533	*p*-Coumaric acid		x			x	
173.0419	134.0375	3-FQA feruloylquinic acid			x			
191.0072	167.9749/155.9433/110.9921	Quinic acid derivative		x				
191.0524	179.0513/113.0108/119.0220/105.0031	Quinic acid			x		x	
311.057	227.9305/179.0267/148.9988/135.0298	Caffeoyl tartrate		x				
315.0549	152.9976/109.0022	Protocatechuic acid glucoside					x	
325.0358	193.0236/135.0131	Ferulic acid pentose derivative		x				
325.1244	163.0275/119.0309	Coumaroylglucose	x					
337.0937	191.0468/163.0406/119.0533	3-*p*-Coumaroylquinic acid	x		x			
337.1012	173.0419	5-*p*-Coumaroylquinic acid	x					
337.1049	191.0496	*p*-Coumaric acid derivative	x					
337.1162	191.0524/163.0197	Coumaroylquinic acid	x					
337.1162	173.0365/163.0249	Coumaroylquinic acid	x					
341.1113	195.0507/163.0249/119.0354	*p*-Coumaric acid derivative	x					
345.1148	193.0521/146.9424	Ferulic acid derivative		x			x	
353.0648	191.0298/147.0367	4′-Caffeoylquinic acid	x		x	x		x
353.0918	191.0666/179.0321/173.0419	Caffeoylquinic acid	x		x	x		
353.1033	191.0439/179.0458	5′-Caffeoylquinic acid	x	x	x	x		x
367.0899	173.0419	4 FQA tri-feruloylquinic acid *trans*			x			
367.0923	193.0464/191.0666	Feruloylquinic acid isomer			x			
367.1002	173.0419	4 FQA tri-feruloylquinic acid *cis*			x			
367.108	191.0524	5 FQA tri-feruloylquinic acid			x			
417.1147	307.0882/187.0564/163.0302/145.0259/119.0533	Coumaroyl-hexose hydroxyphenol						x
417.1231	307.0739/163.0354/145.0284/119.0465	Coumaroyl-hexose-hydroxyphenol						x
433.1075	323.0721/203.0314/179.0294/161.0241/135.0393	Caffeoyl-hexose-hydroxyphenol						x
473.065	311.0389/179.0431/149.0138/135.0440	Caftaric acid and hexose derivative		x				
475.1194	179.0404/161.0293/135.0488	Caffeic acid derivative						x
475.1328	301.0390/179.0349/161.0215	Caffeic acid derivative						x
515.101	353.0802/191.0553/179.0404/173.0419	Dicaffeoylquinic acid				x		
515.1289	353.0802/191.0581/179.0349/173.0446	Dicaffeoylquinic acid				x		
515.2682	191.0637/179.0515	Dicaffeoylquinic acid					x	
591.1038	439.9709/295.0253/179.0075	Caffeic acid derivative		x				
623.078	311.0281/179.0075	Dicaftaric acid		x				
Propiophenone								
327.1203	147.0367	DHPPG (3,4′-dihydroxypropiophenone-3-β-d-glucoside)	x					

The results of quantitative analysis of main phenolics (flavonols, phenolic acids, flavanols, procyanidins), iridoids and DHPPG are shown in Table 3. The lingonberry extract had the greatest amount and variety of phenolics.

**Table 3:** Quantitative analysis of major compounds identified in extracts from plants (mg/100 g dw)

	Flavonols (mg QG/g)	Phenolic acids (mg CQA/g)	DHPPG (mg CQA/g)	Iridoids (mg LA/g)	Flavanols/procyanidins (mg C/g)	Sum of main phenolics (mg/g)
*B. pendula*	92.9	15.4	34.4	Nd	Nd	142.6
*E. arvense*	8.2	19.6	Nd	11.0	Nd	38.8
*G. odoratum*	69.7	53.2	Nd	58.1	Nd	181.0
*H. glabra*	6.9	10.2	Nd	11.8	Nd	28.8
*U. dioica*	Nd	2.1	Nd	Nd	Nd	2.1
*V. vitis*-*idaea*	134.6	10.7	Nd	12.6	41.1	199.1

*QG* quercetin-3-glucoside, *CQA* caffeoylquinic acid, *LA* loganic acid, *C* catechin, *DHPPG* 3,4′-dihydroxypropiophenone-3-β-d-glucoside, *Nd* not detected

### Molecular characterization of bacterial strain

**Fig. 1:**
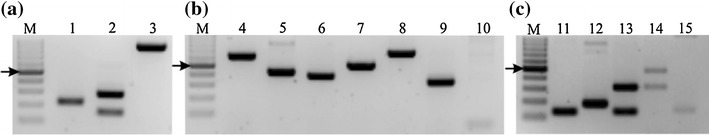
Agarose gel electrophoresis of amplified PCR products. **a** phylogenetic analysis, **b** virulence factors genes, **c** biofilm-related genes. Lanes: *M* molecular size markers (100 bp, Fermentas), *1*—*yjaA*, *2*—*chuA* (*upper band*), *TspE4.C2* (*lower band*), *3*—16SrRNA (control), *4*—*aer*, *5*—*sfa*, *6*—*csgA*, *7*—*cnf1*, *8*—*hlyA*, *9*—*papC*, *10*—*afa*, *11*—*ant43*, *12*—*luxS*, *13*—*sdiA* (*lower band*), *14*—*mqsR*, *15*—*mcbA*. *Arrows* indicate 500 bp. *Upper band* visible on *lane 13* may result from the non-specific amplification of some plasmid-encoded gene or/and *sdiA* rearrangement, since they were not obtained with DNA template from CTF073 strain (data not shown). *Bands* visible on *lane 14* are non-specific

Phylogenetic studies have shown that *E. coli* strains can be divided into four main phylogenetic groups, designated A, B1, B2, and D [16], depending on the presence/absence of two genes *chuA* and *yjaA* and an anonymous DNA fragment *TspE4.C2* [5]. On this basis, our *chuA*-positive and *yjaA*-positive strain belongs to the phylogenetic group B2 (Fig. 1a). The most frequently observed *E. coli* phylogenetic groups among UPEC strains are B2, D and occasionally A, B1 [17]. Thus, the *E. coli* strain used in our research is classified as UPEC.

It is well known that uropathogenic strains possess genes encoding specific virulence factors that play an important role in the pathogenicity by overcoming host defence mechanisms and causing disease. The most common occurring are adhesins P fimbriae (pilus associated with pyelonephritis, *pap*), S fimbriae (*sfa*), afimbrial adhesin (*afa*) and curli fiber (*csgA*), siderophore aerobactin (*aer*), toxins hemolysin (*hlyA*) and cytotoxic necrotizing factor 1 (*cnf1*). As shown on Fig. 1b all of these genes are present in the genome of our UPEC strain except *afa* gene. The occurrence of some biofilm-related genes, namely *ant43*, *luxS*, *sdiA*, and *mcbA* suggests that analyzed *E. coli* strain has the ability to crate biofilm structure (Fig. 1c).

### Antibacterial activity of plant extracts

**Fig. 2:**
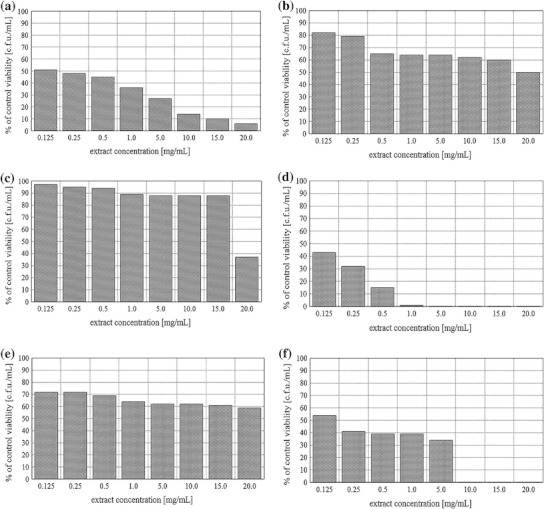
The percentage of *E. coli* strain survival after exposure to: **a** *B. pendula*, **b** *E. arvense*, **c** *G. odoratum*, **d** *H. glabra*, **e** *U. dioica*, **f** *V. vitis*-*idaea* extracts

Results obtained in the present study showed that the tested plant extracts possessed different antimicrobial activities (Fig. 2). The number of bacterial cells (c.f.u/mL) in control sample was 4.5 × 10^9^. Increased concentrations of plant extracts caused decrease in survival of bacterial cells. The extracts of *H. glabra* and *V. vitis*-*idaea* showed the highest bactericidal activity (*p* < 0.05). The strongest inhibition of bacterial growth was observed at *H. glabra* extract concentrations of 1.0 5.0, 10.0, 15.0, 20.0 mg/mL. Very strong reduction of *E. coli* growth was also observed during incubation of bacteria in *V. vitis*-*idaea* extract concentrations of 10.0, 15.0, 20.0 mg/mL. Effect of *B. pendula* extract was slightly less efficient in comparison with *H. glabra* and *V. vitis*-*idaea* extracts. The percentage viability of bacterial cells decreased from 51 % (at concentration of 0.125 mg/mL) to 6 % (at concentration of 20 mg/mL) of the control sample. This was statistically significant (*p* < 0.05). *U. dioica* and *E. arvense* extracts showed similar antimicrobial activity patterns. Exposure of rods to *U. dioica* extracts inhibited their growth from 72 % (at concentration of 0.125 mg/mL) to 59 % (at concentration of 20 mg/mL) of the control sample. The percent of survival of bacteria incubated in the presence of *E. arvense* extracts was reduced from 82 % (at concentration of 0.125 mg/mL) to 50 % (at concentration of 20 mg/mL) of the control. Figure 2 clearly shows that *U. dioica* and *E. arvense* extracts were more effective than *G. odoratum* extract and less effective than *H. glabra*, *V. vitis*-*idaea* and *B. pendula* extracts*. G. odoratum* extract had the weakest antimicrobial activity with the exception of concentration of 20 mg/mL which represents 37 % of the control sample (*p* < 0.05).

### Effect of plant extracts on hydrophobicity of bacterial cells

The results showing the effect of plant extracts on cell surface hydrophobicity are shown in Table 4. Surface hydrophobicity of autoaggregating *E. coli* strain changed after exposure to plant extracts, with the exception of *V. vitis*-*idaea* extract. The cell surface hydrophobicity changes were observed in bacteria treated with *G. odoratum* and *U. dioica* extracts at concentrations of 15.0 and 20.0 mg/mL. Cells surfaces were classified as hydrophilic because they aggregated in 3.2 M ammonium sulfate. This result was not statistically significant (*p* = 0.241). Other concentrations of *G. odoratum* and *U. dioica* extracts resulted in bacteria aggregation in the lower concentrations of ammonium sulfate (0.4–1.0 M) indicating strong hydrophobic bacterial cells surface. Rods incubated in all concentrations of *E. arvense* and *H. glabra* and in 5.0, 10.0, 15.0 and 20.0 mg/mL *B. pendula* extracts showed strong hydrophobic cells surface, because they aggregated in 0.4–1.0 M ammonium sulfate. The lower concentrations of *B. pendula* extracts resulted in bacteria aggregation in 0.1–0.2 M, hence their cell surface was considered to be very strong hydrophobic.

**Table 4:** Effect of plant extracts on hydrophobicity of *E. coli* bacterial cells

Plants	Extract concentrations (mg/mL)								
	Control	0.125	0.25	0.5	1.0	5.0	10.0	15.0	20.0
*B. pendula*	Autoaggregative^#^	Very strong hydrophobic (0.1)^§^	Very strong hydrophobic (0.1)	Very strong hydrophobic (0.1)	Very strong hydrophobic (0.2)	Strong hydrophobic (0.4)	Strong hydrophobic (0.4)	Strong hydrophobic (0.4)	Strong hydrophobic (0.4)
*E. arvense*	Autoaggregative	Strong hydrophobic (0.8)	Strong hydrophobic (0.8)	Strong hydrophobic (0.8)	Strong hydrophobic (0.8)	Strong hydrophobic (0.8)	Strong hydrophobic (0.8)	Strong hydrophobic (0.8)	Strong hydrophobic (0.8)
*G. odoratum*	Autoaggregative	Strong hydrophobic (0.4)	Strong hydrophobic (0.4)	Strong hydrophobic (0.8)	Strong hydrophobic (0.8)	Strong hydrophobic (0.8)	Strong hydrophobic (0.8)	Hydrophilic (3.2)	Hydrophilic (3.2)
*H. glabra*	Autoaggregative	Strong hydrophobic (1.0)	Strong hydrophobic (1.0)	Strong hydrophobic (1.0)	Nt	Nt	Nt	Nt	Nt
*U. dioica*	Autoaggregative	Strong hydrophobic (0.4)	Strong hydrophobic (0.4)	Strong hydrophobic (0.4)	Strong hydrophobic (1.0)	Strong hydrophobic (1.0)	Strong hydrophobic (1.0)	Hydrophilic (3.2)	Hydrophilic (3.2)
*V. vitis*-*idaea*	Autoaggregative	Autoaggregative	Autoaggregative	Autoaggregative	Autoaggregative	Autoaggregative	Nt	Nt	Nt

*Nt* not tested (bacterial survival lower than 5 %)

^#^The strain formed the aggregates in PBS

^§^The lowest molar concentration of (NH_4_)_2_SO_4_ causing visible bacterial aggregation

### Effect of plant extracts on swimming motility

**Fig. 3:**
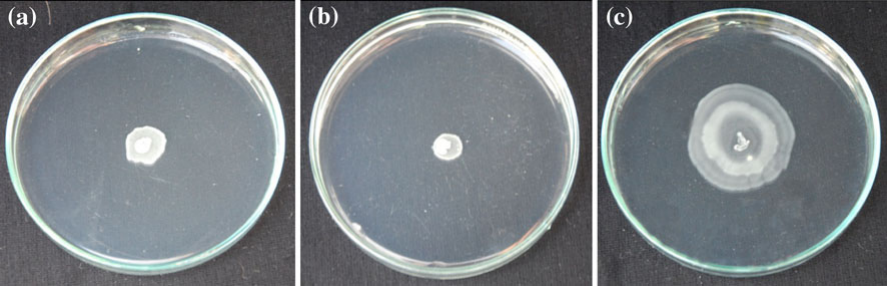
Representative images of *E. coli* swimming motility under control conditions (**a**); in the presence of *U. dioica*—20.0 mg/mL (**b**) and *E. arvense*—10.0 mg/mL (**c**)

The diverse effects of plant extracts on swimming motility of motile *E. coli* strain were recorded (Table 5). The largest concentrations of *B. pendula* (15.0, 20.0 mg/mL) and *U. dioica* extracts (20.0 mg/mL) significantly reduced the motility of the examined strain (*p* < 0.05). Swimming zone diameters ranged between 10.7 (±1.5) and 12.7 (±0.6) mm (Fig. 3b). Bacteria treated with *B. pendula* (5.0, 10.0 mg/mL) and *U. dioica* extracts (5.0–15.0 mg/mL) also showed decreased movement abilities. Motility of bacteria incubated in the presence of *H. glabra* and *V. vitis*-*idaea* extracts decreased slightly compared to the control sample (16.2 ± 2.0). Swimming zone diameters ranged from 14.0 (±2.0) to 16.0 (±2.6) mm. Low concentrations of *B. pendula* and *U. dioica* extracts (0.125–1.0 mg/mL) and all concentrations of *E. arvense* as well as *G. odoratum* extracts did not inhibit bacterial movement. On the contrary, they resulted in increasing of the swimming zone diameter [16.3 (±4.2) to 36.7 (±7.4) mm] (Fig. 3c).

**Table 5:** Effect of plant extracts on *E. coli* swimming motility. Results showing the motility zone are the mean values from three experiments (±SD)

Plants	Extract concentrations (mg/mL)								
	Control	0.125	0.25	0.5	1.0	5.0	10.0	15.0	20.0
*B. pendula*	16.2 (±2.0)	18.7 (±3.1)	18.3 (±1.5)	19.3 (±4.2)	18.7 (±5.5)	15.0 (±1.7)	12.7 (±2.1)	11.7 (±3.1)*	12.7 (±0.6)*
*E. arvense*	16.2 (±2.0)	18.7 (±2.1)	20.3 (±0.6)*	25.0 (±2.6)*	34.3 (±7.5)*	28.3 (±2.9)*	36.7 (±7.4)*	26.7 (±4.2)*	23.0 (±1.7)
*G. odoratum*	16.2 (±2.0)	16.3 (±4.2)	16.3 (±4.0)	21.0 (±3.6)*	17.0 (±3.5)	23.3 (±1.5)*	31.3 (±1.5)*	32.7 (±5.5)*	30.0 (±3.5)*
*H. glabra*	16.2 (±2.0)	15.7 (±4.0)	15.7 (±3.2)	16.0 (±2.6)	Nt	Nt	Nt	Nt	Nt
*U. dioica*	16.2 (±2.0)	21.3 (±3.1)*	17.3 (±2.5)	16.7 (±4.6)	20.0 (±2.0)*	12.7 (±4.2)	15.3 (±2.1)	14.0 (±2.0)	10.7 (±1.5)*
*V. vitis*-*idaea*	16.2 (±2.0)	15.7 (±2.5)	14.3 (±2.5)	14.3 (±0.6)	14.0 (±2.0)	15.3 (±1.5)	Nt	Nt	Nt

*Nt* not tested (bacterial survival lower than 5 %)

* Result is statistically significant (*p* < 0.05)

### Effect of plant extracts on hemagglutination and expression of P fimbriae

The effects of plant extracts on hemagglutination and expression of P fimbriae are shown in Table 6. They depended on the type and concentration of the plant extract used. The lowest extract concentration causing no agglutination was 1.0 mg/mL and it was observed in case of *V. vitis*-*idaea* extract. Bacteria incubated with *G. odoratum* lost their hemagglutination ability at extract concentrations of 5.0 mg/mL and higher. In case of *B. pendula* and *U. dioica*, examined rods failed to agglutinate erythrocytes after exposure to extract concentrations of 10.0, 15.0 and 20.0 mg/mL. Bacteria growing in the presence of *E. arvense* and *H. glabra* extracts did not show any alteration of the analyzed properties.

**Table 6:** Effect of plant extracts on P fimbriae (P) and curli fibers (C) synthesis by *E. coli*

Plants	Extract concentrations (mg/mL)																
	Control	0.125		0.25		0.5		1.0		5.0		10.0		15.0		20.0	
		P	C	P	C	P	C	P	C	P	C	P	C	P	C	P	C
*B. pendula*	+	+	+	+	+	+	+	+	+	+	+	−	+	−	+	−	+
*E. arvense*	+	+	+	+	+	+	+	+	+	+	+	+	−	+	−	+	−
*G. odoratum*	+	+	+	+	+	+	+	+	+	−	+	−	+	−	+	−	+
*H. glabra*	+	+	+	+	+	+	+	Nt	Nt	Nt	Nt	Nt	Nt	Nt	Nt	Nt	Nt
*U. dioica*	+	+	+	+	+	+	+	+	+	+	+	−	+	−	+	−	+
*V. vitis*-*idaea*	+	+	−	+	−	+	−	−	−	−	−	Nt	Nt	Nt	Nt	Nt	Nt

+, present; −, absent

*Nt* not tested (bacterial survival lower than 5 %)

### Effect of plant extracts on curli expression

The impact of plant extracts on the occurrence of the curli fibers is shown in Table 6. Only bacteria incubated in the presence of all concentrations of *V. vitis*-*idaea* extract and 10.0, 15.0 and 20.0 mg/mL of *E. arvense* extracts formed white colonies which indicated the loss of curli fimbriae. Other extracts did not inhibit the synthesis of curli fibers. Bacterial colonies had red color.

### Effect of plant extracts on biofilm formation

The activities of plant extracts at concentration 0.125 mg/mL against total biomass of *E. coli* biofilm are shown in Table 7. In all cases, the strongest and statistically significant biofilm reduction was noticed after 4, 5, and 6 days of bacterial incubation (*p* < 0.05). The amount of biofilm ranged from 1.4 to 2.0 % in comparison to control samples (ODs = 0.0001). Equally strong inhibition of biofilm mass production was observed after 9- and 10-day incubation in *E. arvense* and *H. glabra* extracts and after 10-day treatment in case of *V. vitis*-*idaea* extract (ODs = 0.0001).

**Table 7:** Effect of plant extracts on *E. coli* biofilm formation

Time of incubation (days)	Biofilm formation													
	control		*B. pendula*		*E. arvense*		*G. odoratum*		*H. glabra*		*U. dioica*		*V. vitis*-*idaea*	
	OD (±SD)	(%)	OD (±SD)	(%)	OD (±SD)	(%)	OD (±SD)	(%)	OD (±SD)	(%)	OD (±SD)	(%)	OD (±SD)	(%)
1	0.004 (±0.001)	100.0	0.002* (±0.002)	50.0	0.0001* (±0.000)	2.5	0.0001* (±0.000)	2.5	0.001* (±0.0005)	25.0	0.0001* (±0.000)	2.5	0.0001* (±0.000)	2.5
2	0.005 (±0.002)	100.0	0.004 (±0.003)	80.0	0.0001* (±0.000)	2.0	0.004 (±0.002)	80.0	0.002* (±0.001)	40.0	0.002* (±0.001)	40.0	0.003* (±0.002)	60.0
3	0.01 (±0.002)	100.0	0.01 (±0.003)	100.0	0.002* (±0.0002)	20.0	0.007 (±0.003)	70.0	0.003* (±0.001)	30.0	0.007 (±0.003)	70.0	0.01* (±0.003)	100.0
4	0.005 (±0.002)	100.0	0.0001* (±0.000)	2.0	0.0001* (±0.000)	2.0	0.0001* (±0.000)	2.0	0.0001* (±0.000)	2.0	0.0001* (±0.000)	2.0	0.0001* (±0.000)	2.0
5	0.005 (±0.001)	100.0	0.0001* (±0.000)	2.0	0.0001* (±0.000)	2.0	0.0001* (±0.000)	2.0	0.0001* (±0.000)	2.0	0.0001* (±0.000)	2.0	0.0001* (±0.000)	2.0
6	0.007 (±0.001)	100.0	0.003* (±0.001)	42.8	0.0001* (±0.000)	1.4	0.0001* (±0.000)	1.4	0.0001* (±0.000)	1.4	0.0001* (±0.000)	1.4	0.0001* (±0.000)	1.4
7	0.01 (±0.002)	100.0	0.006* (±0.002)	60.0	0.002* (±0.001)	20.0	0.005* (±0.002)	50.0	0.003* (±0.001)	30.0	0.004* (±0.001)	40.0	0.0001* (±0.000)	1.0
8	0.014 (±0.002)	100.0	0.009* (±0.003)	64.3	0.003* (±0.001)	21.4	0.008* (±0.002)	57.1	0.004* (±0.002)	28.6	0.008* (±0.002)	57.1	0.01 (±0.003)	71.4
9	0.008 (±0.002)	100.0	0.008 (±0.003)	100.0	0.0001* (±0.000)	1.3	0.003* (±0.001)	37.5	0.0001* (±0.000)	1.3	0.003* (±0.001)	37.5	0.007 (±0.0025)	87.5
10	0.005 (±0.001)	100.0	0.005 (±0.003)	100.0	0.0001* (±0.000)	2.0	0.001* (±0.0004)	20.0	0.0001* (±0.000)	2.0	0.002* (±0.001)	40.0	0.0001* (±0.000)	2.0

Results are the mean ODs for 7 experiments

* Result is statistically significant (*p* < 0.05)

The detailed analysis of the obtained results showed the most effective inhibitory effect of *E. arvense* extract. This plant significantly (*p* < 0.05) reduced the optical density (ODs ≤0.003). The maximum amount of biofilm mass was only 21.4 % of the control sample (8th day). Similar strong inhibitory effect was shown for *H. glabra* extract. The largest amount of biofilm mass represented only 40 % of the control.

The weakest effect was demonstrated for *B. pendula* extract. After 3, 9 and 10 days, it did not inhibit biofilm formation. In the remaining days, the amount of biofilm was reduced from 42.8 to 80 %, with the exception of 4 and 5 days, when the synthesis of biofilm mass was strongly inhibited (2 % of the control).

## Discussion

It is known that in the prevention and treatment of urinary tract infections, one should use medicinal herbs as supplement of the daily diet. Cranberry is one of the most recommended plants by both, doctors and pharmacists. Due to its properties, this fruit prevents adhesion of pathogenic bacteria to uroepithelial tissue, what results in inhibition of their growth and multiplication. Apart from cranberry; however, many other plants are used in folk medicine to prevent or to treat bacterial infections. As an example of such plants may serve *B. pendula*, *E. arvense*, *G. odoratum*, *H. glabra*, *U. dioica* and *V. vitis*-*idaea*, which are used in traditional medicine. Many reports describe their medical properties (diuretic, diastolic, diaphoretic activities and anti-inflammatory effect) which are caused by their chemical composition typical for each species. Phytochemical investigations have shown that these plants contain mainly flavonoids, glycosides, saponins, tannins and terpene derivatives [18, 19]. According to the results presented in this paper, *B. pendula*, *E. arvense*, *G. odoratum*, *H. glabra*, *U. dioica* and *V. vitis*-*idaea* extracts should also be remembered and added to the list of herbs which can be used in UTIs. Unfortunately, only a few research groups described the antibacterial activity of birch, horsetail, woodruff, rupturewort, nettle and lingonberry extracts [20–22]. For this reason, the purpose of our study was to determine the effect of these extracts on bacterial survival, virulence factors involved in tissue colonization and biofilm formation.

The findings of the present study clearly indicate that the tested extracts exhibit significant differences in their antimicrobial activities against *E. coli* rods. The susceptibility order was as follows: *H. glabra* > *V. vitis*-*idaea* > *B. pendula* > *E. arvense* > *U. dioica* > *G. odoratum*. Based on the qualitative and quantitative analysis of plant extracts (Tables 2, 3), it can be concluded that the differences in susceptibility of bacterial strains to these extracts do not depend on the content of phenolic compounds. It can, therefore, be suspected that other compounds such as saponins, tannins and terpenes are responsible for the bacterial growth inhibition. Khanna and Kannabiran [23] reported that saponin fractions purified from leaves of some plants show antimicrobial activity against Gram-negative strains: *Pseudomonas aeruginosa*, *E. coli*, *Salmonella typhi*, *Klebsiella pneumoniae*, and *Proteus mirabilis*. Research carried out by Ho et al. [20] and Barile et al. [24] revealed that saponins, terpenes and tannins found in *Vernonia amygdalina*, *Allium minutiflorum* and *V. vitis*-*idaea* constitute the antimicrobial components of these plants. In our experiments, we established that high concentrations of saponin-rich *H. glabra* and *V. vitis*-*idaea* extracts show the strongest bactericidal activity among the rest tested plants. The growth of bacteria was totally inhibited by extracts concentrations ranging from 1.0 to 20.0 mg/mL for *H. glabra* and 10.0–20.0 m/mL in case of *V. vitis*-*idaea*.

In contrast to our above-presented results, Kylli et al. [21] found that phenolic proanthocyanidins-rich extracts of lingonberries had antimicrobial effect only on Gram-positive, but not on Gram-negative bacteria: *Salmonella enterica* sv. Typhimurium, *Lactobacillus rhamnosus* and *E. coli*. This result shows that separately isolated compounds do not always have to be as effective as multi-component extracts. Saric et al. [25] examined the influence of *B. pendula* ethanolic extract on the growth of different bacterial species and found that the concentration 10 mg/mL of this extract possesses the strongest antimicrobial effect against *Bacillus cereus*. This plant exhibited moderate antimicrobial activity against the other investigated bacteria. Njume et al. [26] examined several different extracts of *Combretum molle* against *Helicobacter pylori*. Their results have shown the significant correlation between bacterial survival and the extracts concentration and the type of solvent used in the extraction process.

Looking at the results obtained in our investigation (Fig. 2), *B. pendula* extract should be forcefully considered as an efficient *E. coli* multiplication inhibitor and hence as agent reducing the bacterial survival. All tested concentrations of this extract significantly decreased bacterial growth. The least effective extracts were those derived from *U. dioica* and *G. odoratum* leaves. Even their highest used concentrations (20.0 mg/mL) in this study did not fully inhibit bacterial growth but only reduced their survival. The viability level was 59 % for bacteria treated with *U. dioica* and 37 % in case of *G. odoratum* compared to the control samples. Similar to the above-presented results, Singh et al. [21] established no activity either of *U. dioica* aqueous or methanol and ethyl-acetate extracts against Gram-positive *Staphylococcus aureus*, as well as Gram-negative *Shigella flexneri*, *P. aeruginosa*, *K. pneumoniae* and *Salmonella typhi*. The chloroform extract of common nettle showed only moderate action against tested microorganisms, while good antibacterial properties against all studied bacterial species were found for hexane extract of *U. dioica*. These results clearly show that the antibacterial abilities of the extract strongly depend on the reagents used during its extraction.

The limited number of publications describing the influence of plant extracts on bacterial virulence factors prompted us to perform research in this area. The most important pathogenic factors involved in bacterial adhesion to uroepithelial cells are hydrophobic surface, the ability to movement and synthesis of adhesins.

In our study, the changes in the cell surface properties were observed for *E. coli* incubated in *G. odoratum* and *U. dioica* extracts. Very strong hydrophobic cells’ surface of autoaggregative *E. coli* strain used in our experiments became hydrophilic after exposure only to the highest concentrations (15.0 and 20.0 mg/mL) of both extracts. Hydrophilic cell surface nature impedes the colonization of the host tissues. Therefore, despite the weak growth-inhibiting properties, *G. odoratum* and *U. dioica* can be applied in UTIs prevention. Similar results of bacterial hydrophobicity changes have been noticed by Razak et al. [27]. The authors established that the hydrophobicity of *Streptococcus mitis*, *Streptococcus sanguinis* and *Actinomyces* sp. incubated in the presence of *Psidium guajava* extract was reduced.

The analysis of the swimming zone diameters obtained in our research showed that some of the extracts have reduced, and other have increased the bacterial motility. This phenomenon is difficult to explain, because there are no reports describing similar experiments. Probably, this effect is associated with different pH values of the plant extracts. Hattermann and Ries [28] found that bacteria growing at pH 6 and 7 were more motile than those grown at pH 5.8–10.0. Hidalgo et al. [29] showed that swimming and swarming motilities were hindered when *E. coli* CFT073 strain was grown in the presence of the cranberry compounds. This result was due to inhibition of flagellin gene (*fliC*) expression. Transmission electron microscopy imaging of bacteria exposed to cranberry materials revealed fewer flagella than in control bacteria.

The presence of fimbrial adhesins promotes the attachment of the bacterial cells to the host tissues and protects them against removing from the urinary tract with urine. We established that extracts of silver birch, sweet woodruff, common nettle and lingonberry inhibited erythrocyte hemagglutination by uropathogenic *E. coli* strain, which indicates the dysfunction of P fimbriae. It is known that these plants are rich in tannins—compounds with the structure very similar to receptors found on bladder and kidney cells [30]. Therefore, these compounds act by binding to fimbriae and thereby preventing their attachment to the host tissue. Ahuja et al. [31] found that *E. coli* rods growing in the presence of the cranberry juice lost the expression of P fimbriae leading to a loss of the ability to epithelial cells colonization. Proanthocyanidins present in cranberry fruits are responsible for this phenomenon. Strong inhibition of adherence of multi-drug resistant *E. coli* strains treated with proanthocyanidins to uroepithelial cells was also observed by Gupta et al. [32]. Curli fibers play an important role in biofilm formation by rods belonging to Enterobacteriaceae family [33]. It has been also shown that the reduction of pili correlated with the loss of the ability of uropathogenic *E. coli* strains to colonize bladder cells and to form biofilm [34]. In our study, bacteria growing in all tested concentrations of *V. vitis*-*idaea* extracts and the highest concentrations of *E. arvense* extracts (10.0–20.0 mg/mL) showed no expression of curli fimbriae.

According to some researchers, recurrent UTIs are caused by microorganisms that invade the urinary tract and form a biofilm structures [35]. The results of our study indicate that the exposure of *E. coli* rods to plant extracts significantly reduced or inhibited the biofilm production. Such activity of these plant extracts can be explained by the presence of flavonoids. It is known that flavonoids such as quercetin, kaempherol, naringenin and apigenin reduce biofilm synthesis because they can suppress autoinducer-2 activity which is responsible for cell-to-cell communication [36]. Lee et al. [37] confirmed that phloretin belonging to flavonoids suppressed autoinducer-2 importer genes of *E. coli* O157:H7 biofilm cells.

The results obtained in our study suggest that anti-biofilm effect of plant extracts can be caused by modifications in the bacterial surface structures responsible for binding to the occupied surface.

## Conclusion

All plant extracts used in our study showed antibacterial activity and/or reduction of the biofilm mass. Only some extracts altered virulence factors in examined rods. Therefore, our results should be confirmed in clinical trials to be able to recommend tested plant extracts in prevention of and treatment of UTIs. Moreover, considering the wide variety of the antibacterial activities of plant extracts, depending on the solvent, it would be worthwhile in the future studies to compare our current results with, e.g., methanol- or ethanol-derived extracts.
